# Combined Experimental and Theoretical Insights into the Corrosion Inhibition Activity on Carbon Steel Iron of Phosphonic Acids

**DOI:** 10.3390/molecules26010135

**Published:** 2020-12-30

**Authors:** Aurelia Visa, Nicoleta Plesu, Bianca Maranescu, Gheorghe Ilia, Ana Borota, Luminita Crisan

**Affiliations:** “Coriolan Dragulescu” Institute of Chemistry, 24 M. Viteazul Ave, 300223 Timişoara, Romania; avisa@acad-icht.tm.edu.ro (A.V.); bmaranescu@acad-icht.tm.edu.ro (B.M.); ilia@acad-icht.tm.edu.ro (G.I.); ana_borota@acad-icht.tm.edu.ro (A.B.)

**Keywords:** corrosion, phosphonic acids, computer modeling, simulation

## Abstract

The inhibition effect of *N*,*N*′-phosphonomethylglycine (PMG) and vinyl phosphonic acid (VPA) on the 3% NaCl acidic solution corrosion of carbon steel iron was studied at different immersion times by potentiodynamic polarization, electrochemical impedance spectroscopy, attenuated total reflectance Fourier transform infrared (ATR-FTIR) spectroscopy, and computational methods. It is found from the polarization studies that PMG and VPA behave as mixed-type inhibitors in NaCl. Values of charge transfer resistance (R_ct_) and double layer capacitance (C_dl_) in the absence and presence of inhibitors are determined. The PMG and VPA inhibitors were capable of inhibiting the corrosion process up to ≈91% and ≈85%, respectively. In the presence of PMG, the synergic effect of chlorine ions was observed. Density functional theory (DFT) was engaged to establish the adsorption site of PMG, VPA, and their deprotonated states. For studied compounds, the resulted values of *E*_LUMO_, *E*_HOMO_, energy gap (*∆E*), dipole moment (*μ*), electronic hardness (*η*), global softness (*σ*), electrophilic index (*ω*), and the electronic potential map are in concordance with the experimental data results regarding their corrosion inhibition behavior and adsorption on the metal surface.

## 1. Introduction

In recent years, many researchers have been focused on the development of inhibitors for environmental safety purposes. Various organic compounds were successfully applied as corrosion inhibitors. These compounds are containing heteroatoms such as N, O, and P in their molecules. In this regard, nitrogen-containing structures such as Schiff bases [1,2], imidazole, triazole, benzimidazole [3,4], phosphonates, metal organic frameworks [5,6,7,8], phosphonic acids [9,10,11], and solid polymer electrolytes based on phosphorus [12,13,14] have been studied as corrosion inhibitors for iron in acid media.

Organic compounds containing heteroatoms as N, P, O, and/or *p*-electrons or non-bonding electrons demonstrate good results for the inhibition of corrosion. The heteroatom inhibitory efficacy decreases in the order: P > N > O. Phosphonates and phosphonic acids are well known as inhibitors capacity with a negligible impact on the environment in water treatment. This class of compounds can adsorb almost instantaneously on the metal surfaces and form a protective layer. This behavior reduces the exposure area of the active metal to the corrosive environment.

Corrosion inhibition efficiency correlates with the structure of phosphonate-based molecules [15,16,17]. Kálmán and coworkers studied the corrosion inhibition for a low-concentration of 1-hydroxyethane-1,1-diphosphonic acid (HEDP) and high concentration of HEDP on neutral carbon steel. The HEDP inhibits the carbon steel corrosion at low concentration via a precipitation mechanism; thus, the higher concentration of HEDP decreases the inhibitory efficiency due to the dissolution of the oxide layer [18].

Organic compounds with phosphorus can cover many application areas such as sensors, corrosion assay materials, cooling water, electro-optics, filtration, ion exchange, and as catalysts [15]. These compounds are resistant to hydrolysis and high temperatures and show good corrosion inhibition properties [19]. The studies have shown that the efficiency of corrosion (IE) is closely related to their structures. Their efficiency arises from their capacity to bind (phosphonic acid group) in a monodentate, bidentate, or tridentate manner to a metal surface [7,16,20,21]. Therefore, they can act both as mineral scale and corrosion inhibitors [22,23]. Phosphonates such as diphosphonic acids, aminotris-(methylenephosphonic) acid (ATMP), diethylenetriamine-pentamethylenephosphonic acid (DTPMP), hydroxyphosphono acid (HPA), 1-Hydroxyethane-1,1-diphosphonic acid (HEDP), *N*,*N*–dimethylidenephosphonoglycine (DMPG), 1–ethylphosphonoethylidenediphosphonic acid (EEDP), of nitrilotrimethylenephosphonic acid (NTMP), and ethylenediamine tetramethylidenephosphonic acid (EDTMP) were inspected for protecting mild steel from corrosion [18,24,25,26,27]. An efficient inhibitor seems to be the phosphonic acids that pose both N and O atoms (as in the ATMP molecule), which are both capable of coordinating with Fe^2+^ in the film. The thiomorpholin-4-ylmethyl-phosphonic acid (TMPA) and morpholin-4-methyl-phosphonic acid (MPA) offer corrosion protection of carbon steel in natural seawater [28]. Tetraphosphonates with six or eight carbon atoms on the chain generate a well-organized packing on the carbon steel surface, while the two carbons in tetraphosphonate form a thin, incomplete, and porous layer on the carbon steel surface [21]. 

Valuable information can be extracted from the frontier orbitals that allowed us to investigate their tendency to form a protection film on the metal surface [29,30]. The literature mentioned the corrosion assay of 2-phosphate group-1,2,4-tricarboxylate butane (PBTCA), hydroxyethylidene disphosphonic acid (HEDP), and aminotrimethylene phosphonic acid (ATMP) and reported that the corrosion inhibition rate of these three organic phosphorus reagents can be presented in the following order BTCA > HEDP > ATMP. Corresponding to the molecular layout analysis and the frontier orbital theory, these three organophosphorus were used as electron buffers. They are capable of receiving the free electron of the outer orbit of the iron atom and forming a stable coordination bond. The protective film formed on the metal surface was further capable of obstructing the metal corrosion. In addition, the inhibitor can provide electrons to zinc ions present in the water, forming complex compounds, and permit safe water circulation in the cooling system [31].

Therefore, the present study is focused on two phosphorous acids and their corrosion inhibition of iron in NaCl solution. The inhibition property offered by these acids was investigated electrochemically, and the quantum chemical method was employed to calculate the molecular structure parameters and to explore the mechanism of corrosion inhibition. Theoretical studies were undertaken to offer molecular level information on the corrosion inhibition action of investigated materials on iron surfaces and to additional support experimental data.

## 2. Materials and Methods

### 2.1. Experimental

All chemicals were of reagent grade quality achieved from commercial sources and used without further purification. *N*,*N*-bis(phosphonomethyl)-glycine (PMG) and vinylphosphonic acid (VPA) were purchased from Merck (Milipore, Darmstadt, Germany), and NaCl, HNO_3_ 65% were purchased from Sigma Aldrich Chemie GmbH (München, Germany). Low-carbon steel specimen contains chemical composition (wt%) of C: 0.20%, Mn: 0.45%, P: 0.04%, S: 0.05% and Fe balance, were made from an iron bar, which was purchased from EPI Sistem (Brasov, Romania).

#### 2.1.1. Potentiodynamic Polarization (CP) Testing and Electrochemical Impedance Spectroscopy (EIS) Measurements

Electrochemical tests: electrochemical impedance spectroscopy (EIS) spectra and polarization curves (CP) were performed to assess the corrosion inhibitory effect of PMG and VPA in 3% NaCl solution, adjusted with HNO_3_ (65%) to pH ≈ 2.3 (The pH was measured using a pH meter (Mettler Toledo, Giessen, Germany)). The control electrolyte solution was 3% NaCl with pH ≈ 2.3. A Dcorr cell corrosion cell and Autolab 302N EcoChemie (Netherlander, 2007) was employed. The working electrode was a coupon carbon steel with an exposed area of 0.785 cm^2^. Two graphite bars and Ag/AgCl were used as counter electrodes and the reference electrode, respectively. The experimental data were used to estimate the corrosion rate (*CR*) and electrochemical parameters such as corrosion potential—*Ecorr*, corrosion density current—*Jcorr,* and polarization resistance—*Rp* of carbon steel in NaCl 3% solution, at pH ≈ 2.3 from Tafel plots. EIS measurements were conducted at open circuit potential (OPC) values, at 22 ± 1 °C under potentiostatic mode using ac signals of amplitude 10 mV peak to peak in the frequency range from 10.000 to 0.01 Hz. The experimental electrochemical impedance data were fitted to the electrical equivalent circuit (EEC) by a complex non-linear least squares Levenberg–Marquardt procedure using ZView 3.0 software (Scribner Associates, Inc., Southern Pines, NC, USA). The EIS and CP tests were performed after one and two hours of immersion. All the experiments were conducted in triplicate.

#### 2.1.2. Attenuated Total Reflectance Infrared (ATR)

All spectra were recorded with an FT/IR-4200 JASCO Spectrophotometer, equipped with PIKe ATR (MIRacle), DTGS detector, Ge crystal plate with a resolution of 4 cm^−1^ in the range of 4000–600 cm^−1^. All data were analyzed by the Spectral Manager Version 2 software.

#### 2.1.3. Optical Microscopy

The Zeiss Stemi 508 microscope (CarlZeiss Microscopy GmbH, Gottingen, Germany) was used to investigate the morphology of the films formed on the surface of the carbon steel.

### 2.2. Theoretical Chemical Calculations

The molecular structures of VPA, PMG, and their mono and bi deprotonated species at phosphonic moiety group VPA^1−^, VPA^2−^, PMG^1−^, PMG^3−^, and PMG^4−^ were pre-optimized using the MM+ (Molecular Mechanics) Force Field and further refined using the semi-empirical PM3 (Parametric Method 3) method included in HyperChem 7.52 (Hypercube, Inc., Gainesville, FL, USA) package [32]. Geometry optimization was achieved by setting a Polak–Ribere algorithm with an SCF (Self Consistent Field) convergence limit of 0.00001 kcal/mol, and an RMS (Root-Mean-Square) gradient norm limit of 0.01 kcal/(Å·mol). The complete geometric optimization of the PM3 geometries was carried out with a Jaguar module of the Schrödinger package by applying density functional theory (DFT) incorporating the B3LYP-D3/6-31G** basis set [33,34]. Furthermore, for both neutral and the mono and bi deprotonated structure of VPA and PMG, frequency calculations were performed to check if there are true minima. The lack of any negative frequencies certified the true energies minima of the compounds. To portray the anticorrosion action of VPA and PMG, their lowest energy conformations were selected to further calculate several quantum chemical descriptors such as the lowest unoccupied molecular orbital (LUMO) and the highest occupied molecular orbital (HOMO), the ionization potential (*IP*; Equation (1)), the electron affinity (*EA*; Equation (2)), the energy gap (*ΔE*; Equation (3)), hardness (*η*, Equation (4)), softness (*σ*; Equation (5)), and electrophilicity index (*ω*; Equation (6)). The results of the calculated descriptors provide key information about the electronic structure, conformation, and reactivity of VPA and PMG compounds, which support their anticorrosive action.

(1) $$IP=\;-\;E_{HOMO}$$

(2) $$EA=\;-\;E_{LUMO}$$

(3) $$\Delta E=\;\;E_{LUMO}-\;E_{HOMO}$$

(4) $$\eta =IP-EA=\frac{E_{LUMO}-E_{HOMO}}{2}$$

(5) $$\sigma =\;\frac{1}{\eta }$$

(6) $$\omega =\;\frac{\mu^{2}}{2\eta }$$

For the compound that shows the highest probability of adsorption on the metal surface, the electrostatic potential was displayed. This parameter depicts the interaction between the compound and a positive point charge (in our study, Fe^2+^). It is very useful for identifying sites of reaction in a compound. It is expected that molecule where the electrostatic potential is strongly negative to be susceptible to an electrophilic attack.

## 3. Results

### 3.1. Potentiodynamic Polarization Study 

The polarization measurement results were used to evaluate the protective properties of inhibitors on the corrosion of carbon steel in NaCl solution at a pH = 2.3, at two different film-forming times: 1 h and two hours. Potentiodynamic polarization measurements were carried out by scanning the electrode potential from −1200 to −200 mV (vs. Ag/AgCl) with a scan rate of 0.01 mV·s^−1^.

**Figure 1:**
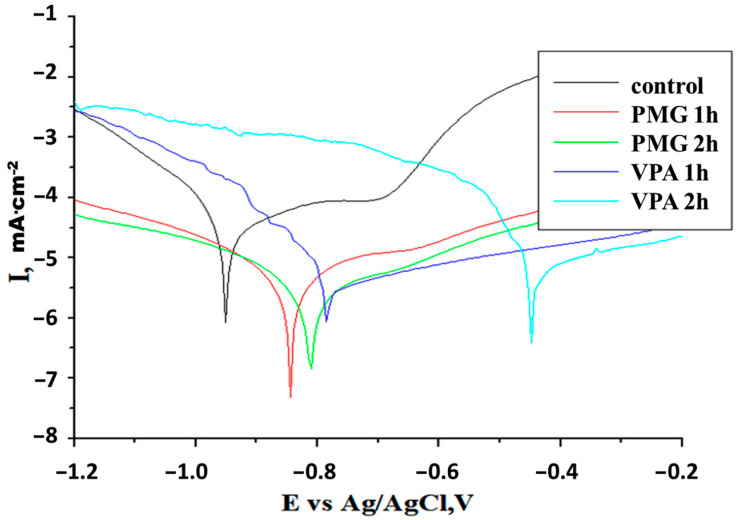
Potentiodynamic polarization curves for carbon steel after different immersion times in 3% NaCl solutions, pH = 2.3 without (control) and with 2 mM inhibitor (scan rate = 1 mV/s), at 22 °C.

Figure 1 displays the potentiodynamic polarization curves recorded in an acid medium of 3% NaCl, which were obtained before and after the introduction of 2 mM inhibitors (this concentration was used based on previous results) [8]. Various corrosion parameters (cathodic—*βc* and anodic—*βa* Tafel slope, corrosion potential—*Ecorr*, corrosion current density—*Jcorr*, polarization resistance—*Rp*, and corrosion rate—*CR*) were determined from Tafel plots at different immersion times. The values are summarized in Table 1. 

These values obtained from the extrapolation of anodic and cathodic Tafel lines located next to the linearized current regions represent the mean value of three determinations. 

It can be observed that the *Jcorr* values for all inhibitors (Table 1) were lower than those for the “control”. The higher anodic currents for iron indicated that the iron suffers corrosion via its dissolution into ferrous and ferric cations. The differences observed for the values of *Jcorr* and *Rp* indicate better protection of carbon steel in the presence of PMG. In the presence of PMG, the lowest *Jcorr* and highest *Rp* were obtained: ≈5.76 × 10^−6^ A·cm^−2^ and 6.733 × 10^3^ Ohm·cm^−2^ after 1 h and 3.67 × 10^−6^ A·cm^−2^ and 1.363 × 10^4^ Ohm·cm^−2^ after 2 h of immersion. Experimental data show that the protection offered by the tested inhibitors is maintained over time and changes due to the adsorption of molecules at the metal surface. As the immersion time increases, the corrosion currents decrease, and the *Rp* increases in the case of PMG and decreases slightly in the case of VPA. Also, the values of the corrosion rate, *CR* was lower in the presence of PMG. The anodic polarization curves are shifted to positive values for all inhibitors. The values of cathodic and anodic Tafel slopes suggest some modification in the inhibition corrosion mechanism due to the adsorption process and to the formation of a protective layer at the active metal surface. 

The differences obtained in the values of Tafel slopes indicate a different way by which the inhibitors are absorbed on the metal surface. Inhibitors are adsorbed at the metal/solution interface and are capable to block the active site, providing more protection to the metallic surface. The corrosion potential (*Ecorr*) is shifted to a more noble value (positive value) compared with that of the bare metal. 

The shape for the anodic and cathodic parts suggests that the addition of inhibitors reduced the hydrogen evolution and oxidation of the metal. The addition of inhibitors affected anodic and cathodic reactions therefore these inhibitors can be considered as mixed-type inhibitors with an anodic prime effect. 

For the VPA, the *βa* is smaller than *βc*, implying that the electrode reaction of the carbon steel surface is mainly controlled by cathode reduction. In the case of the PMG, the *βa* is larger than *βc* as the electrode reaction is most probably a mixed controlled one. 

Based on the corrosion current density data, the inhibition efficiency was calculated according to Equation (7):

(7) $$IE=\frac{J_{corr}-J_{inh}}{J_{corr}}\times 100$$

where *IE* represents the inhibitory efficiency expressed in %, and *Jcorr* and *Jinh* are the corrosion current density without and with inhibitor, respectively.

PMG and VPA offer a good inhibition efficiency with max values of 91% and 86% respectively, as a result of the formation of a passivating film on the metallic surface. It is clear that the surface coverage (θ) and inhibition efficiency (*IE*) varies with immersion time and depends on the inhibitor nature. At this concentration (2 mM) of inhibitor with the increase of immersion time, the adsorption of inhibitor molecules take place, and more inhibitor molecules will be adsorbed onto the iron surface, which is capable of forming and maintaining the protective layer. The differences observed with the increase of immersion time are the result of the reorientation of inhibitor molecules and/or their adsorption/desorption from the surface.

### 3.2. ATR Spectral Studies 

**Figure 2:**
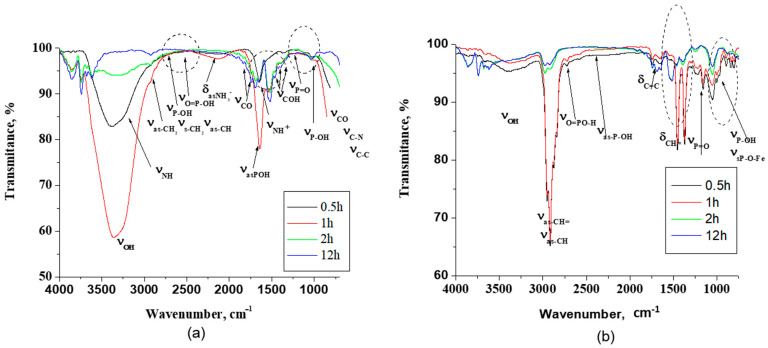
ATR-FTIR spectra for (**a**) *N*,*N*′-phosphonomethylglycine (PMG) and (**b**) for vinyl phosphonic acid (VPA).

The ATR spectral studies were conducted to investigate the protective film formed on the metal surface by the inhibitor molecules. The spectra are presented in Figure 2a,b.

The assignment of bands for PMG presents difficulties, due to the presence of some regions with very broad and structured bands as -NH^+^, -P=O, -P-OH, -COO^−^, and -COOH, groups are present in solution (Figure 2a). In the ATR spectra of the film formed in the presence of PMG in 3500–2800 cm^−1^ frequency domain, very broad bands were observed that reflect a highly complex spectral shape most probably due to intermolecular and/or intramolecular hydrogen bonding formed by -PO_3_H_2_, NH^+^, COH, and the COOH groups.

The film formation was confirmed by the disappearance of the hydrogen bond between the inhibitor molecules, which confirms the bond formation between the inhibitor and the metal surface during the adsorption process [35]. The bands at 3255 cm^−1^ were attributed to ν_as NH_^+^ and at 3212 cm^−1^ to ν_sNH_^+^. The CH_2_ and CH stretching modes ν_sCH2,_ ν_asCH2,_ and ν_sCH_ appear as a shoulder at 2945 and at 2927 cm^−1^ and at 2905 cm^−1^, respectively. The weak broad absorption at near 2700 cm^−1^ was attributed to P-O-H [36]. The bands at 2550 cm^−1^ and at ≈2190 cm^−1^ were associated with O=P-OH vibrations. The phosphonate group vibrations (-PO_3_H_2_) were attributed to the bands at 1643 cm^−1^, at 1225 cm^−1,^ and at 1100–1140 cm^−1^ due to ν_asP-OH_, ν_P=O_, and ν_P-OH_ stretching mode, respectively [37,38]. 

The carboxyl group presents bands due to the carbonyl and alcohol group vibrations. The bands at 1819 cm^−1^ and 1790 cm^−1^ were assigned to the ν_C=O_ stretching mode. The main -COH bending modes are grouped at 1466, 1415, and 1383 cm^−1^. The -NH^+^ group stretching modes are expected to appear not only above 3200 cm^−1^ but at ≈1600 cm^−1^ associated with δ_NH_^+^ and to C-NH rocking deformation and wagging bond vibrations, and they overlapped with the COH and -PO_3_H_2_ groups. These bands were attributed mainly to the ν_sO=P-OH_ absorption band, but it is possible for this to overlap with the ν_sCOO_^−^, δ_asNH_^+^ vibration and δCH_2_ absorption bands. The range 900–1200 cm^−1^ is a complex spectral region characteristic of vibrations related to the -PO_3_ moiety. Bands at 1050, 929, and 902 cm^−1^ correspond to the ν_asP=O_ bond, C-N bond stretching, and C-C skeletal stretching [39]. The band at 1038 cm^−1^ was attributed to a zwitterionic structure involving intramolecular hydrogen bonding between P-O^−^ and NH^+^, which is an assumption in agreement with results found for glyphosate [40].

The differences in the vibration frequencies of the O=P-OH are probably due to the participation of this group in the formation of hydrogen bonds with different strengths. The phosphonic group can bind to the iron via a direct P-O-Fe bond in different modes, i.e., mono, bi-, or tridentate, as one, two, or all three oxygen atoms from the phosphonic group are involved [41]. The ATR spectra reveal a time-dependent adsorption process. It is observed that the carboxylate bands (Figure 2a, middle marked zone) occur in these films at frequencies and an intensity similar to PMG. This suggests a weak carboxylate interaction with a metallic substrate. 

Decreasing the intensity and shifting to the lower wavelength of the characteristic absorption band at 3388 cm^−1^ related to the OH stretching mode of the molecules shows that as the immersion time increases, the number of free OH groups in films decreases, and they can participate in the formation of intermolecular hydrogen bonds. Moreover, the appearance of a new, broad absorption band at prolonged immersion time at 2550 cm^−1^ associated with O=P-OH…O sustains also the formation of hydrogen bonds (Figure 2a, highlighted zone) [38]. 

The decrease of the band at ≈1600 cm^−1^ attributed to the asymmetric bending band of the -NH^+^ also proves that the nitrogen atom is also involved in the adsorption process, through the formation of a complex. With the increase of immersion time, the characteristic band at 1225 cm^−1^, which corresponds to the P=O stretching mode, decreases as the PMG molecules self-assembled onto the iron substrates by a multidentate binding (lower gap energy indicated by computational studies) [42].

The phosphonate band at 1100–1140 cm^−1^ in PMG is assigned to P-O^−^ stretching motions (ν_P-O_^−^). Its absence in the corresponding films indicates the multidentate binding by all three oxygens of phosphonate group (Figure 2a, highlighted). Moreover, the corresponding band for films appears at lower wavenumber (1050 cm^−1^) characteristic for deprotonated species. This suggests further interaction of the P=O moiety and the bond formation with the iron surface. This observation is corroborated by the higher Rct attributed to strong bond formation at 2 h of immersion time and computational result. In consequence, it is possible to assume that the interaction between the amine group and Fe surface brings the PO_3_^2−^ groups closer to the metal surface. This assumption is supported also by the high degree of surface coverage and by the high Δ*G*°*_ads_* value. 

The ATR spectra of carbon steel immersed in VPA reveal the film formed on the surface absorption peaks linked to the bonds P=O, -P-OH, and C=C respectively (Figure 2b). 

The O-H, -C-H, CH=, and C=C bands appear at 3400–3200 cm^−1^, at 2940–2900 cm^−1^, and at 1678–1600 cm^−1^, respectively. The band assigned to PO_3_^2−^ appears approximately as in the case of PMG as follows: at 3340 cm^–1^, it was assigned to the O-H stretching band; at 2900 cm^–1^, it was assigned to C-H stretching vibration; at 2700 cm^−1^, it was assigned to the stretching of O=P-O-H; at 2333 cm^–1^, it was assigned to P-OH stretching; at 1350 cm^–1^ and 1255 cm^−1^, it was assigned to the stretching of P=O; and at 980 cm^–1^, it was assigned to the O-P-O band and/or to P-OH. The literature data mentioned that the low intensity of the bands attributed to the P-OH stretching mode and the presence of PO_3_^2−^ stretching modes (deprotonation of the phosphonic acid group) in the spectrum is an indication for a bidentate binding [43], which is observed also in our case: a lower gap energy indicated by computational studies. Other researchers conclude that the bidentate binuclear complex takes place at high pH and low surface coverage, whereas the protonated monodentate mononuclear complex is dominant at low pH and high surface coverage [44]. It is more probable that in the film formed in the presence of VPA, both monodentate and bidentate complexes are present (Figure 2b). The increase of P-OH and P=O stretching bands with the increase of the immersion time reveal a decreasing tendency of a multidentate bind. With the increase of surface coverage, the binding modes can turn to a monodentate binding. Even the desorption of this acid is possible. As a consequence, R_ct_ and IE will decrease. The band at 1692 cm^−1^ was attributed to C=C stretch. The position of the C=C stretching frequency varies slightly as a function of orientation around the double bond and is less informative than the information brought by vibrations of CH=. The vibration CH= out-of-plane and in-plane bending appear at 1420 cm^−1^ and 968 cm^−1^, respectively. The decrease of these bands with the increase of immersion time also suggests some hydrogen bonding or deprotonation processes that dominate the adsorption of VPA at the metal surface, which are responsible for reactions that decrease the strength of the formed protective layer, as shown in Figure 2b in the highlighted zones.

### 3.3. Electrochemical Impedance Spectroscopy (EIS) Measurements

**Figure 3:**
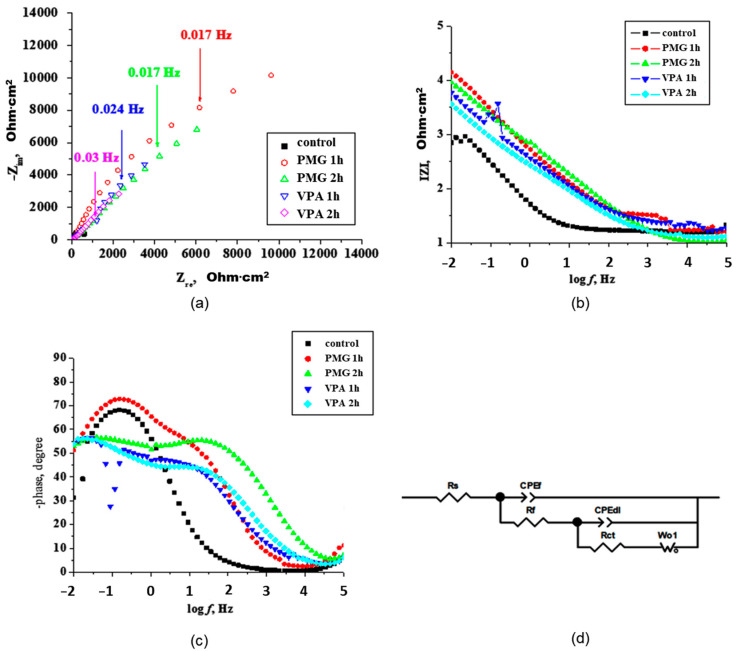
(**a**) Complex plane Nyquist plots, (**b**) Bode plots the logarithm of the impedance modulus |ZI, (**c**) phase angle as a function of the logarithm of the frequency *f* for iron immersed for 1 h in 3.5% NaCl solutions in the presence of inhibitors and (**d**) electrical equivalent circuit.

Impedance spectra represented in both complex impedance diagrams (Nyquist plot) and Bode are illustrated in Figure 3. In the Nyquist graph (Figure 3a), the imaginary component of the impedance is plotted as a function of the real component. The Bode representation displays the logarithm of the impedance modulus |Z| (Figure 3b) and phase angles as a function of the logarithm of the frequency *f* (Figure 3c).

Figure 3a reveals an increase in the capacitive arc radius by adding inhibitors in the solution. The PMG and VPA present a similar form for capacitive arcs. The arcs are non-ideal semi-circles due to the frequency dispersion caused by the corrosion of metal and/or adsorption on inhibitors at the metal surface [45]. The kinetic parameters were obtained by fitting with an appropriate equivalent circuit (Figure 3d) and the resulting parameters are summarized in Table 2. The electrical circuit used consists of solution resistance—R_s_, film resistance—R_f_, charge transfer resistance—R_ct_, and a diffusional element W (Warburg element). The capacitive elements used, CPE_f_ and CPE_dl_, represent the constant phase element of the film and the electrical double layer, respectively. 

In the Bode phase plots (Figure 3c), two-time constants are present. The loop observed at low frequency (LF) was attributed to the mass transfer and that at the intermediate frequency was linked to the R_ct_ and double layer capacitance, as was reported by other authors [46]. The incomplete capacitive loop observed at high frequency is due to the non-homogeneous current distribution on the electrode surface. In the intermediate frequency region, the highest value for the phase angle is observed for PMG at 2 h (≈60°). The phase angle, in the case of PMG, shows an increasing tendency with the increase of immersion time. For VPA, the phase angle values are lower and remain almost constant with the increase of immersion time. In the presence of inhibitors, the charge transfer resistance (R_ct_) increases and the double-layer capacitance (C_dl_) decreases as a result of the adsorption of the organic molecules at metal surfaces [47]. Instead of a pure capacitance, a CPE element was used. The CPE represents the deviation from ideal dielectric behavior due to surface heterogeneity. This element consists of two elements, the exponent (CPE-P) and the pseudocapacitance (CPE-T). The capacitance can be calculated with Equation (8) [48]:

(8) $$Z_{CPE}=\frac{1}{T{\left(J.\omega \right)}^{\phi }}$$

where 0 < *φ* < 1 describes the deformation of the circle in the complex plane and is a constant. If *φ* = 1, CPE becomes a perfect capacitor. *ω* is the angular frequencies (in rad·s^−1^, with *ω* = 2π·f), *f* is the frequency (in Hz). 

The T parameter is proportional to the capacity of the double layer, as shown in Equation (9):

(9) $$T=C_{ds}^{\varphi }{\left(R_{s}^{-1}+A\right)}^{1-\varphi }$$

where *C*${}_{ds}^{\varphi }$ = capacity of the double layer, in F; *R_s_* = solution resistance, in Ω; *A* = electrode surface area, in cm^2^.

The best fits obtained by modeling EIS data with equivalent electrical circuit presented in Figure 3c are presented in Table 2.

Table 2 shows that the R_ct_ and C_dl_ evolve in time as the composition of the inhibitor layer changes. The results show a higher resistance R_f_ value for PMG both at 1 h and 2 h. The inhibitor VPA presents lower values of resistance R_f_ both at 1 h and 2 h. This suggests that PMG adsorb and form a compact layer on the metal surface. This compact layer contains a low number of ionically conducting paths and presents a higher corrosion performance. The VPA layer seems to be more porous. The high number of ionically conducting paths present in the VPA layer will allow corrosion species to be present on the metal surface. The CPE_f_ values decrease with immersion time. The decrease is associated with the adsorbed layer, which can become thicker, or to the decrease in the dielectric constant of the protective layer (Equation (10)).

(10) $$C=\frac{\varepsilon_{r}\varepsilon_{0}A}{d}$$

where *C* represents the capacitance, *ε*_0_—void permittivity, 8.8 × 10^−12^ F·m^−1^, *ε_r_*—dielectric constant of the protective layer, *A*—area in cm^2^, and *d*—thickness of the coating (layer).

In all determinations, CPR_f_ decreased in time, as the protective layer evolved. The CPE_f_ in the case of VPA is lower than in the presence of PMG. This means a thicker protective layer formed by the VPA inhibitor, but corroborated with the R_f_ value, the layer is porous.

The lower R_ct_ value obtained for VPA suggests a lower ability of this acid to link to the metal surface and to form a porous layer that in time can become incomplete and easy for corrosive species to penetrate. These results are sustained by adsorption data, CP data, and ATR. The addition of inhibitors to the solution also modifies the double-layer capacitance C_dl_. A lower value of CPE_dl_ means good adherence. Higher R_ct_ and lower C_dl_ values are obtained for PMG, indicating the formation of a thicker and compact layer. The bonding through P-O ionic species and a nitrogen atom on the iron surface in a stronger manner leads to a more efficient packing on the surface (confirmed also by ATR data). Even thought VPA has a strong tendency to utilize the P-OH, P=O, and C=C moieties to form a thicker layer on the metal surface, the majority of bonds are weaker (P-OH-O-Fe) (confirmed also by ATR data). The CPE_dl_-P value gives a measure of the deviation from ideal capacitive behavior, which depends strongly on the state of the surface [48]. The obtained CPE_dl_-P exponents are ranging between 0.42 and 0.74, which are the values that indicate the presence of a non-homogenous surface, especially at prolonged immersion time [49,50]. The calculated time constant τ_d_ (τ_d_ = C_dl_·R_ct_) suggests some differences in the adsorption process with immersion time. The adsorption process becomes slower for higher τ_d_. The results obtained for the time constants show that the lowest value is obtained for the PMG sample at 3.14 s and 2.69 s for 1 h and 2 h, respectively comparing with 6.23 s and 3.35 s at 1 h and 2 h, respectively for the VPA sample. If the layer presents a higher number of pores and is thin, the corrosion process takes place deeper within the pores, close to the metal surface. The displacement of the diffusion-controlled process will take place in this case to lower frequencies. The displacement is more evident with the increase of immersion time. The shifts to lower frequencies are more evident in the case of VPA. In time, the layer becomes more porous, and diffusion phenomena appear. The value of the Warburg impedance coefficient, W-R, represents the resistance originating from the diffusion process through the pores within the corrosion layer. The Warburg impedance was necessary for the fitting of the experimental data process only at prolonged immersion time for both inhibitors. This shows the appearance of resistance due to the diffusion processes as the porosity of films increases over time. The inhibition efficiency (IE) in and surface coverage was calculated from the values of charge transfer resistance by Equations (11) and (12).

(11) $$IE=\frac{R_{ct}^{inh}-R_{ct}^{control}}{R_{ct}^{inh}}\times 100$$

(12) $$\Theta =\frac{IE}{100}$$

where *R_ct_^inh^* is the charge transfer resistance for the electrode in the presence of an inhibitor; *R_ct_^control^* is the charge transfer resistance for the electrode in solution without an inhibitor. 

The percentage of inhibitory efficiency calculated based on EIS data reveal the same tendency observed in the values from the polarization data. The highest results are for PMG (90–95%) and they are a little lower for VPA (75–90%). The IE is comparable with the values reported for other phosphate layers [51,52].

### 3.4. Adsorption Isotherm

The adsorption isotherm is considered a good method to express quantitatively the adsorption process and interactions between the adsorbed inhibitor molecules and metal surface and is a helpful factor in the understanding of the inhibition mechanism [53]. The most appropriate adsorption mode can be determined from the covered fraction of the metal surface by adsorbed molecules (*θ*). The values can be determined with Equation (12) from impedance EIS or CP data. Figure S1 (Supplementary Materials) shows the plots for (*C/θ*) in the function of *C_inhibitor_*. where *θ* is the surface coverage, *C* = *C_inhibitor_* is the inhibitor concentration, and *K_ads_* is the adsorption equilibrium constant. 

The linear dependence and the value of the regression coefficient (*R*^2^ = 0.998) show that the Langmuir adsorption isotherm is the best-fit isotherm, as shown in Equation (13).

(13) $$\frac{C}{\theta }=\frac{1}{K_{ads}}+C$$

These inhibitors obey Langmuir adsorption isotherm as other phosphonic acids on the metal surface [54]. The *K_ads_* represents the adsorption strength between the inhibitor and metal surface. More efficient adsorption implies a larger value for *K_ads_* and a smaller value for the dimensionless separation factor, *R_L_* (Equation (14)) [55].

(14) $$R_{L}=\frac{1}{1+K_{ads}C}$$

The highest *K_ads_* value was obtained for PMG. The calculated values of *R_L_* for the inhibitors (Table 3) are less than unity. The values indicate a favorable adsorption process. The free energy of adsorption (Δ*G*°*_ads_*) was calculated using Equation (15):

(15) $$\Delta {G^{\circ }}_{ads}=-RTLn\left(55.5K_{ads}\right)$$

where *R* is the universal gas constant (8.314 J·mol^−1^·K^−1^) and *T* is the absolute temperature in Kelvin, *K_ads_* is the adsorption equilibrium constant, Δ*G*°*_ads_* is the standard free energy of adsorption, and 55.5 is the concentration of water in the solution in mol·dm^−3^.

Generally, a value of Δ*G*°*_ads_* around −20 kJ·mol^−1^ or more positive indicates physisorption, while values around −40 kJ mol^−1^ or more negative point to a chemisorptions [56]. The negative ∆*G*°*_ads_* values obtained and high values of *K*_ads_ (Table 3) show that in the presence of these inhibitors, the adsorption process is spontaneous. In addition, covalent/electrostatic interaction exists, and the degree in surface coverage was developed according to the Langmuir adsorption isotherm.

The value is higher for PMG. The ATR spectra reveal that the phosphonic group undergoes deprotonation upon adsorption, which is more evident in the first hour of immersion. With the increase of immersion time, there is a more compact adsorbed layer due to a bidentate linkage. The absence of a phosphonate peak assigned to P-O stretching motions (ν_P-O_) indicates the absence of uncomplexed P-O. This observation is corroborated by the higher R_ct_ value obtained at a higher immersion time. In consequence, it is possible to assume that the interaction between the amine group and Fe surface brings the PO_3_^2−^ groups closer to the metal surface, and the disappearance of a band attributed to asymmetric bending of the quaternary ammonium ion proves that the carboxylate and nitrogen atom is also involved in complex formation. This is sustained by the value of ∆*G*°*_ads_*, which is a value that indicates combined physisorption and chemisorptions [57]. 

The capacity of VPA to interact to the iron surface through the double bond, P=O, and P-OH groups are responsible for a better surface immobilization, as confirmed by ATR and ∆*G*°*_ads_*. The obtained value around 23 kJ·mol^−1^ suggests for the VPA an adsorption predominant by physisorption. The bond associated with physisorption and/or desorption is weaker and the occurrence of larger pores takes place with the increase of immersion time. 

### 3.5. Surface Analysis

The surface of the carbon steel electrode was achieved at different immersion times between 0.5 and 12 h in 3% NaCl solution without and with inhibitors. Figure S2 (Supplementary Materials) shows all specimens for comparison.

The optical images show that the carbon steel surface was covered substantially by corrosion products in the case of iron immersed only in NaCl solution at pH = 2.3. Comparing the surface of metal immersed in NaCl solution with and without inhibitor, it is obvious that a protective layer on the metal surface was formed in the presence of inhibitors. The formed layer presents a different pattern, depending on the inhibitor and immersion time. The surface formed in the presence of the VPA inhibitor seems less smooth and thinner, suggesting the formation of a porous or less compact film on the metal surface. The surface roughness influences the carbon steel’s corrosion behavior. The surface measurements by optical microscope showed an improvement in the surface smoothness in the occurrence of the corrosion inhibitor, as shown in Figure S3 (Supplementary Materials). This is a result of the adsorption of inhibition. The surface roughness Ra parameter (average roughness) was determined from the arithmetic average of the gray level [58]. 

The arithmetic average of the gray level can be expressed as shown in Equation (16)):

Ra = (∑(|g1 − gm| + |g2 − gm| + ….. + |gn − gm|)/n(16)

where g1, …. and gn are the gray level values of a surface image along one line.

The mean of the gray values (gm) is determined as shown in Equation (17):

gm = (∑(g1 + g2 + ….. + gn))/n(17)

The values of the average roughness (Ra) for the carbon steel surface before and after electrochemical measurements are for PMG: 9.14 μm at 0.5 h, 11.02 μm at 1 h, and 14.87 μm at 2 h. For VPA, they are 18.14 μm at 0.5 h, 16.59 μm at 1 h, and 27.33 m at 2 h. For Fe corroded in 3% NaCl solution without inhibitor, the surface roughness was higher due to the corrosion process: 69.93 μm at 0.5 h, 91.08 μm at 1 h, and 108.17 μm at 2 h. The increase of smoothness in the case of PMG reveals a better performance (Figure S3a from Supplementary Materials). ATR studies also supported the presence of the adsorbed inhibitor on the C-steel surface. An increase in charge transfer resistance (R_ct_) values with decreasing roughness for mild steel tested in ammonium chloride (NH_4_Cl) solution was reported also [59]. The negative value of the adsorption energy for both inhibitors proved that the adsorption process takes place spontaneously. The order of binding energy (PMG > VPA) confirmed the order of the experimental data.

### 3.6. Theoretical Chemical Calculations

**Figure 4:**
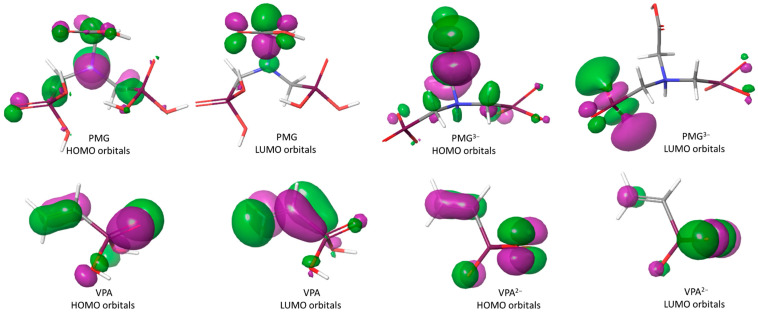
Highest occupied molecular orbital (HOMO) and the lowest unoccupied molecular orbital (LUMO) of PMG, PMG^3−^, VPA, and VPA^2−^ compounds.

Stereochemical characteristics of a molecule can affect its stability and reactivity to other molecules. In this context, the global reactivity descriptors deliver relevant information about compound stability and reactivity. All electronic parameters were calculated based on full optimized geometry (Table 4 and Figure 4). The frontier molecular orbitals, LUMO and HOMO, are valued as key indicators for the chemical reactivity and stability of a molecule. The electron-donating capacity of a molecule to a proper acceptor with empty molecular orbitals (e.g., metal) is expressed by *E*_HOMO_, while the electron-accepting ability is indicated by *E*_LUMO_. The influences of these descriptors on the ability of the anticorrosive compounds to react with a substrate are listed in Table 4.

In addition to the HOMO and LUMO frontiers orbitals, the energy gap is also an essential parameter to explain the activity of an anticorrosive compound. The low value of the energy gap for PMG compared to VPA indicates that it absorbs quickly because a small gap implies less excitation energy to remove electrons from the last occupied orbital. In addition, the dipole moment is an important electronic parameter and has a significant role in corrosion inhibition [60]. The higher value of the dipole moment for PMG (5.2866) comparing with the value of the dipole moment for VPA (1.6265) probably increases the adsorption between PMG and the metal surface [61]. As can be seen in Table 4, the lower values of *E*_LUMO_ show that PMG, followed by VPA, can easily receive free electrons from the metal. Moreover, PMG has the highest *E*_HOMO_ value as compared to VPA. For PMG, the HOMO orbitals appear mainly in the proximity of nitrogen and carbon atoms bound to nitrogen, and of oxygen atoms (=O) bond to phosphorus atoms, while for VPA, they occur in the vicinity of oxygen atoms and the vinyl unit (Figure 4). The unoccupied d orbital of the Fe atom can accept electrons from PMG and VPA to form a coordination bond. PMG and VPA can also accept electrons from the iron atom with its anti-bonding orbitals to form a back-donating bond. These atomic sites assist the adsorption of the PMG and VPA on the metal surface. Additionally, the potential (*μ*), electronic hardness (*η*), electrophilicity index (*ω*), and global softness (*σ*) parameters as complementary information for chemical reactivity of both molecules were evaluated. The concordance with the experimental information was found to be satisfactory.

The same quantum chemical calculations and the same protocol have been successfully applied for the deprotonated species (Table 4 and Table S1 from Supplementary Materials). The PMG^3−^ has the lowest of the energy gap and the highest softness (*σ*). The electrophilicity index (*ω*) also indicates the electron-accepting ability of a compound surface [62]. The value for this descriptor confirms that PMG^3−^ has the highest ability to accept electrons among the studied compounds. These results lead to the conclusion that the adsorption between PMG^3−^ and the metal surface is in all likelihood the highest. This conclusion for PMG^3−^ anticorrosion efficiency is also supported by the electrostatic potential [63], which is pictured in Figure S4. The red color indicates the most negative region while the blue color stands for the most positive region in PMG^3−^ (Figure S4).

The region with a deficiency of electrons (red) is concentrated around the oxygen atoms from PO_3_ groups, which suggests that they represent potential centers of adsorption. The same conclusion emerges by Mulliken charge population analysis (Figure S4). Similar to other investigations [1,2,60,61,62,63], our conclusions highlight the importance of theoretical and experimental insight on the corrosion inhibition behavior for different organic compounds.

Thus, the most stable ionic structure is the deprotonated one at both P-OH from each PO_3_H_2_ group and with NH^+^ called PMG^3−^ and for the other VPA^2−^.

The HOMO–LUMO differences are presented in Table 4. It is observed that for the PMG^4−^ form (PMG^3−^ with COOH deprotonated), the HOMO–LUMO difference is very large (Table S1 from Supplementary Materials). So, the mechanism and the discussion of ATR are in accordance with the most stable ionic forms. From all data, it is revealed that the COOH moiety does not participate in the surface binding.

### 3.7. Mechanism of Corrosion Inhibition

**Figure 5:**
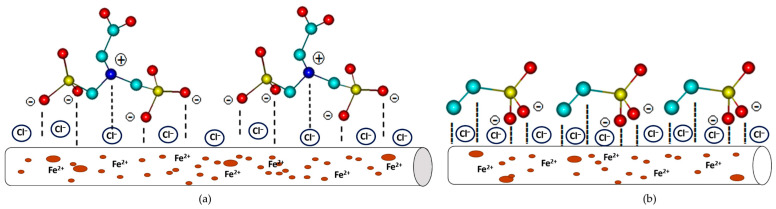
The possible mechanism of (**a**) PMG^3−^ and (**b**) VPA^2−^ corrosion protection studies. Color code: yellow—P, red—O, turquoise—C. The hydrogen atoms are omitted for clarity.

The action of PMG and VPA in saline solution at pH = 2.3 reveals that the studied phosphonic acids are capable of inhibiting the corrosion by controlling both the anodic and cathodic reactions. In acidic solutions, the inhibitors may exist as protonated species. These protonated species adsorb on the cathodic sites of the carbon steel and decrease the evolution of hydrogen. The decreases of anodic reaction can be achieved by adsorption on anodic sites occurs through the π-electrons of a double bond or by the lone pair electrons of nitrogen and oxygen atoms [64]. However, the VPA inhibitory value is slightly lower than the PMG, which may suggest that the nitrogen atom brings an additional contribution to the corrosion inhibition in a significant manner. The PMG molecules present in the solution can be in the form of zwitter ions, in neutral form, or protonated. Lone pairs of electrons of the O and N atoms agree to a stable interaction with the surface (Fe). Coordination through π-electrons of P=O and lone pairs of electrons to the d-orbitals of the Fe surface takes place as a result from experimental data and is in accordance with computational data. At pH ≈ 2.3, a fraction of PMG molecules can have a positive charge as a metal surface that is also positively charged. The repulsive force tends to make adsorption impossible. However, the experimental data show high values of R_ct_ and surface coverage degree. Chloride ions adsorbed on the metal surface will create an excessive negative charge at the metal–solution interfaces, as can be seen in Figure 5.

As a result, PMG^3−^ can be easily adsorbed. Chloride ions show a synergic effect. A similar conclusion was reported in the literature [51,65,66]. The synergic effect of chlorine ions indicated that a small amount of the PMG inhibitor was capable of inhibiting the corrosion process up to ≈97%.

## 4. Conclusions

PMG and VPA were examined to determine their potential to act as a corrosion inhibitor 3% NaCl pH = 2.3 by polarization, impedance, ATR, and computational analysis. The resulting polarization curves suggest that PMG and VPA act as mixed-type inhibitors. Results evidenced that all inhibitors showed excellent performance (IE more than 85%). It was shown that PMG exhibits better protection comparing with VPA at the same concentration. The adsorption process follows the Langmuir isotherm, and it indicates combined physisorption and chemisorption. The different self-assembling processes take place at the metal surface, with the increase of immersion time simultaneously difficult to separate (adsorption and reorientation of molecules). The ATR spectra reveal for PMG a bidentate linkage with the P-OH and nitrogen atom involved in complex formation on the metal surface. This is sustained by the value of ∆*G*°*_ads_*, which indicates combined physisorption and chemisorption for PMG. The capacity of VPA to interact to the iron surface through the double bond, P=O, and P-OH groups are responsible for immobilization, as confirmed by ATR and ∆*G*°*_ads_*, with predominant physisorption. The synergic effect of chlorine ions indicated that a small amount of the PMG inhibitor was capable of inhibiting the corrosion process up to ≈97%. The experimental data and the results of the theoretical calculations are strongly correlated. The calculated values for the *E*_LUMO_ and *E*_HOMO_, gap energy (*∆E*), dipole moment (*μ*), electronic hardness (*η*), global softness (*σ*), electrophilic index (*ω*), and the electronic potential map support the experimental data which indicate that PMG as PMG^3-^ has the best potential anticorrosion efficiency. These results gained in the present study are very useful and can be a basis in the development of more feasible efficient corrosion inhibitors. Molecular modeling studies supported well the experimental data.

## Figures and Tables

**Table 1:** Electrochemical parameters for carbon steel maintained in 3% NaCl solution pH = 2.3 with different inhibitors at 22 °C.

Sample/*SD	*Jcorr*, A·cm^−2^	*Ecorr*, V	*Rp*, Ohm·cm^−2^	*βa*, V/dec	−*βc*, V/dec	*CR*, mm·year^−1^	*IE*, %
control	4.31 × 10^−5^ ± 1.24 × 10^−6^	−0.923 ± 0.032	5.321 × 10^2^ ± 0.272 × 10^2^	0.305 ± 0.032	0.136 ± 0.008	5.016 × 10^−1^ ± 1.021 × 10^−2^	-
PMG 1 h	5.76 × 10^−6^ ± 0.85 × 10^−7^	−0.828 ± 0.021	6.733 × 10^3^ ± 5.653 × 10^2^	0.288 ± 0.021	0.243 ± 0.013	6.009 × 10^−2^ ± 2.191 × 10^−4^	86.627 ± 2.042
PMG 2 h	3.67 × 10^−6^ ± 1.30 × 10^−8^	−0.811 ± 0.054	1.363 × 10^4^ ± 6.003 × 10^2^	0.290 ± 0.009	0.267 ± 0.024	4.271 × 10^−2^ ± 2.323 × 10^−4^	91.485 ± 0.532
VPA 1 h	6.16 × 10^−6^ ± 0.05 × 10^−7^	−0.744 ± 0.082	7.740 × 10^3^ ± 1.732 × 10^2^	0.650 ± 0.002	0.333 ± 0.003	7.171 × 10^−2^ ± 6.24 × 10^−4^	85.703 ± 1.003
VPA 2 h	5.96 × 10^−6^ ± 1.18 × 10^−7^	−0.577 ± 0.067	2.404 × 10^3^ ± 0.834 × 10^2^	0.613 ± 0.004	0.329 ± 0.006	6.941 × 10^−2^ ± 1.71 × 10^−3^	86.162 ± 0.934

*SD—standard deviation.

**Table 2:** Values of the electric circuit elements for the electrodes after 1 and 2 h of immersion.

Sample/*SD	Chi-Sqr	R_s_, Ω·cm^2^	CPE f, F/cm^2^/s·φ ^−1^	CPE-P f, φ	R_f_, Ω·cm^2^	CPE_dl-_T, F/cm^2^/s·φ ^−1^	CPE_dl_-P, φ	R_ct_, Ω·cm^2^	W-R, Ω·cm^2^	*IE*, %
Fe	1.00 × 10^−3^	16.64 ± 1.23	1.60 × 10^−3^ ± 0.29 × 10^−5^	0.72 ± 0.04	19.72 ± 2.32	2.19 × 10^−3^ ± 2.76 × 10^−4^	0.78 ± 0.03	688 ± 31	-	-
PMG 1 h	1.59 × 10^−3^	2.33 ± 0.25	6.75 × 10^−9^ ± 1.08 × 10^−10^	0.76 ± 0.02	38.13 ± 3.09	4.66 × 10^−4^ ± 0.62 × 10^−5^	0.74 ± 0.04	6736 ± 105	-	89.79 ± 0.21
PMG 2 h	1.75 × 10^−4^	2.489 ± 0.19	1.87 × 10^−14^ ± 3.01 × 10^−15^	0.89 ± 0.02	36.67 ± 4.23	1.94 × 10^−4^ ± 0.35 × 10^−5^	0.59 ± 0.03	13,890 ± 223	0.13 ± 0.91	95.05 ± 0.07
VPA 1 h	4.18 × 10^−3^	1.67 ± 0.03	4.85 × 10^−9^ ± 2.54 × 10^−10^	0.69 ± 0.04	34.19 ± 4.12	8.25 × 10^−4^ ± 0.57 × 10^−5^	0.65 ± 0.04	7056 ± 92	-	90.25 ± 0.03
VPA 2 h	1.03 × 10^−3^	1.976 ± 1.06	4.32 × 10^−14^ ± 1.27 × 10^−15^	0.45 ± 0.03	22.104 ± 1.75	1.38 × 10^−3^ ± 0.87 × 10^−5^	0.42 ± 0.07	2829 ± 86	324.2 ± 0.41	75.68 ± 0.23

*SD—standard deviation.

**Table 3:** Adsorption parameters (*K_ads_*, *R_L_* and Δ*G*°*_ads_*) for PMG and VPA.

Inhibitor	PMG	VPA
*K_ads_*, mol^−1^	34676.66	22888.95
∆*G*°*_ads_*, J/mole	−35851.75	−34821.81
*R_L_*	0.13	0.18

**Table 4:** The calculated electronic properties of the studied compounds.

Descriptors		VPA	VPA^2−^	PMG	PMG^3−^
*E*_HOMO_	eV	−7.756	−8.172	−6.373	−12.980
*E*_LUMO_		−0.690	−2.622	−0.577	−7.665
Ionization potential, *IP*		7.756	8.172	6.373	12.980
Electron affinity, *EA*		0.690	2.622	0.577	7.665
Energy gap, *∆E*		7.066	5.550	5.796	5.315
Chemical hardness, *η*		3.533	2.775	2.898	2.657
Electrophilic index, *ω*		2.524	5.248	2.083	20.050
Softness, *σ*		0.283	0.360	0.345	0.376

## Data Availability

The data presented in this study are available on request from the corresponding author.

## Supplementary Materials

The following are available: Figure S1. The isotherm Langmuir plots for tested inhibitors, Figure S2. Optical images for unprotected (control) and protected (by the PMG and VPA) carbon steel specimens, Figure S3. The histogram of surface roughness (a) for PMG and (b) VPA after exposure different time to NaCl 3% solution at pH = 2.3, Figure S4. The electrostatic potential and Mulliken charge distribution in PMG^3−^, Table S1. The calculated electronic properties of the deprotonated compounds.
