# Supplementary material for: Combined Experimental and Theoretical Insights into the Corrosion Inhibition Activity on Carbon Steel Iron of Phosphonic Acids
Source: Molecules. 2020 Dec 30;26(1):135. doi: 10.3390/molecules26010135 (PMC7795472; doi:10.3390/molecules26010135)
Supplement: Supplementary file 1 [file molecules-26-00135-s001.pdf]

molecules

MDPI

Supplementary Information

A combined experimental and theoretical insights into the corrosion inhibition activity on carbon steel iron of phosphonic acids

Aurelia Visa, Nicoleta Plesu*, Bianca Maranescu, Gheorghe Ilia, Ana Borota and Luminita Crisan*

”Coriolan Dragulescu” Institute of Chemistry, 24 M. Viteazul Ave, Timişoara - 300223, Romania; avisa@acad-icht.tm.edu.ro (A.V.); bmaranescu@acad-icht.tm.edu.ro (B.M.); ilia@acad-icht.tm.edu.ro (G.I.); ana_borota@acad-icht.tm.edu.ro (A.B.)

* Correspondence: nplesu@acad-icht.tm.edu.ro (N.P.); lumi_crisan@acad-icht.tm.edu.ro (L.C.)

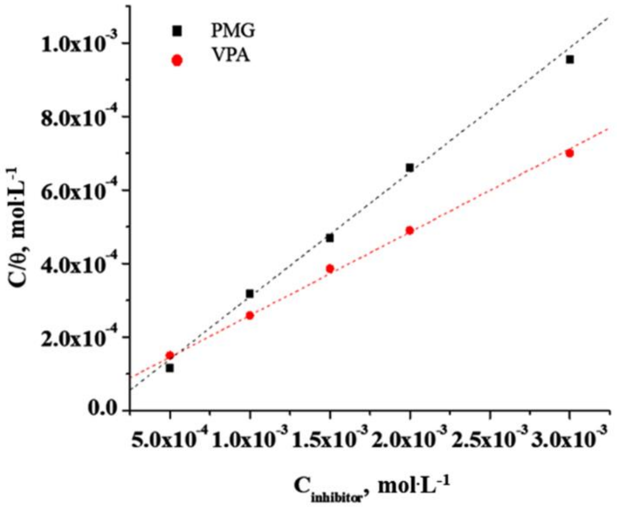

Figure S1. The isotherm Langmuir plots for tested inhibitors.

molecules

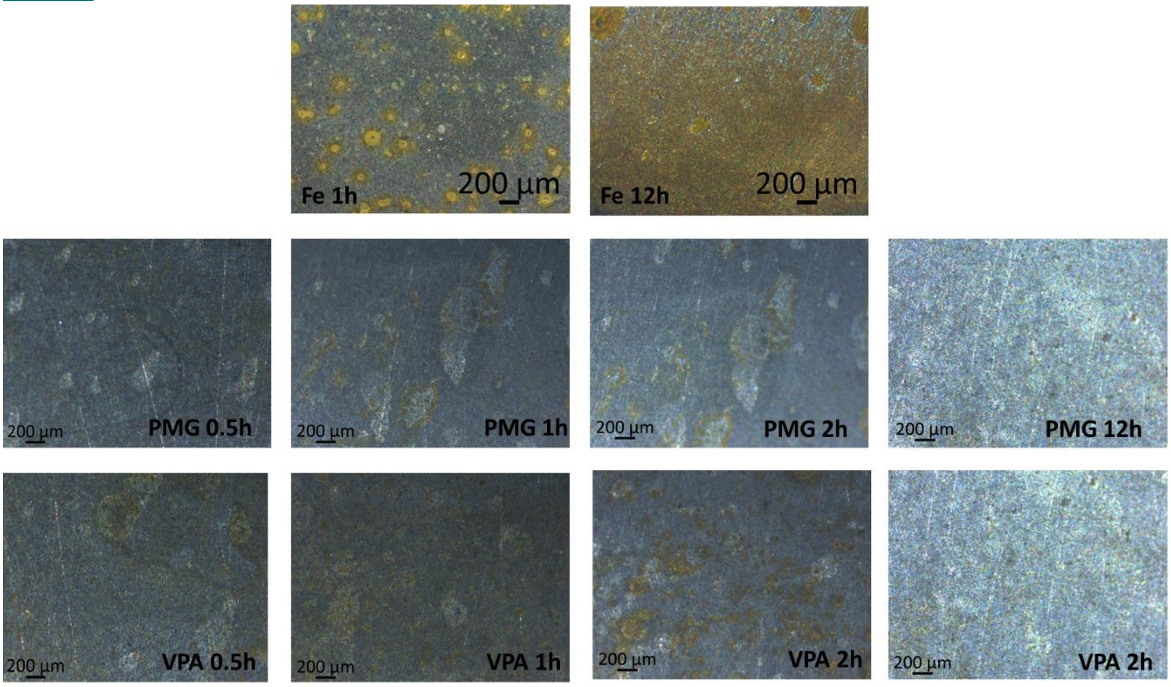

Figure S2. Optical images for unprotected (control) and protected (by the PMG and VPA) carbon steel specimens.

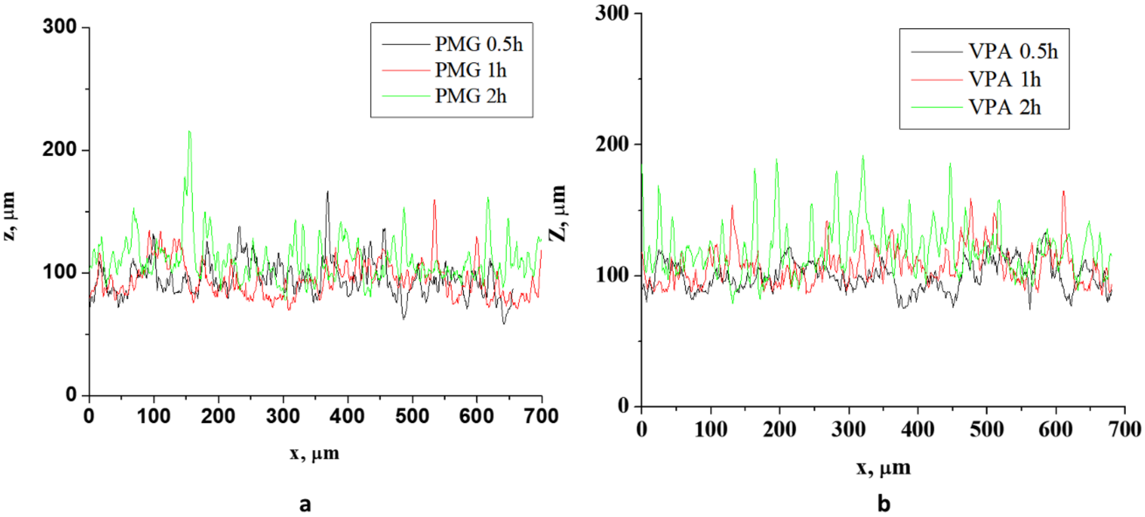

Figure S3. The histogram of surface roughness (**a**) for PMG and (**b**) VPA after exposure different time to NaCl 3% solution at pH = 2.3.

molecules

Table S1. The calculated electronic properties of the deprotonated compounds.

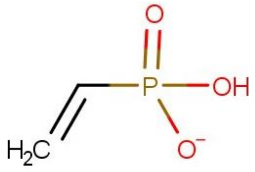

VPA1–

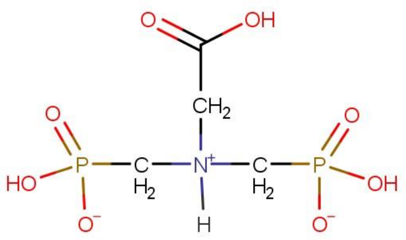

PMG1–

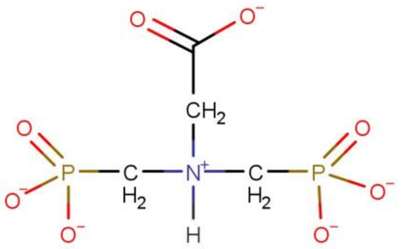

PMG4–

Descriptors		**VPA1–**	**PMG 1–**	**PMG 4–**
E_{HOMO}		−8.063	−12.669	−8.460
E_{LUMO}		−0.544	−6.539	−2.747
Ionization potential, *IP*		8.063	12.669	8.460
Electron affinity, *EA*	eV	0.544	6.539	2.747
Gap Energy, ΔE		7.518	6.130	5.713
Chemical hardness, η		3.759	3.065	2.857
Electrophilic index, ω		2.464	15.047	5.494
Softness, σ		0.266	0.326	0.350

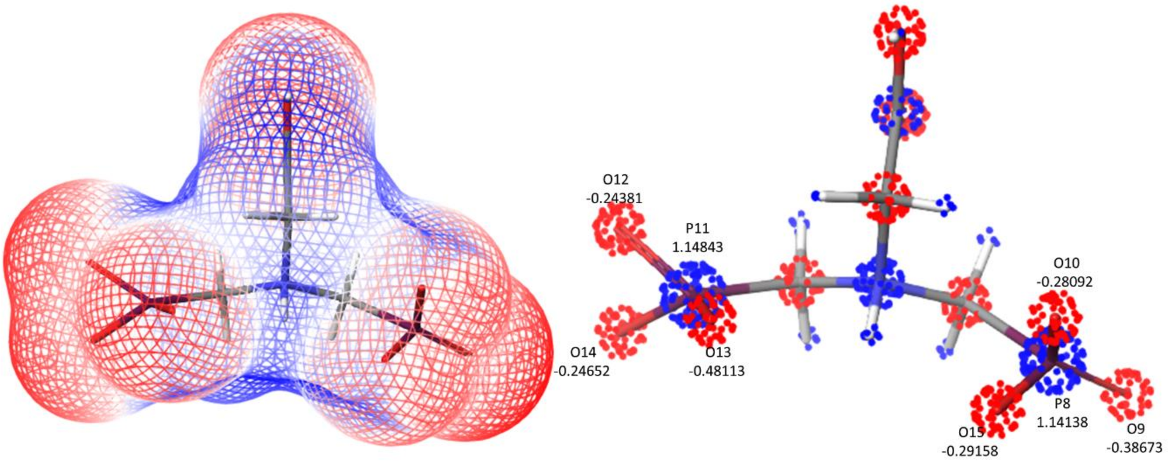

Figure S4. The electrostatic potential and Mulliken charge distribution in PMG^{3-}.
